# Experimental Studies of Microchannel Tapering on Droplet Forming Acceleration in Liquid Paraffin/Ethanol Coaxial Flows

**DOI:** 10.3390/ma13040944

**Published:** 2020-02-20

**Authors:** Jinsong Zhang, Chao Wang, Xianfeng Liu, Chunming Yi, Z. L. Wang

**Affiliations:** 1 School of Mechatronic Engineering and Automation, Shanghai University, No.149 Yanchang Road, Shanghai 200072, China; zhangjs02@163.com (J.Z.); wangchao1994@shu.edu.cn (C.W.); liuxianfeng1992@sina.com (X.L.); yichunming188@163.com (C.Y.); 2 Shanghai Key Laboratory of Mechanics in Energy Engineering, Shanghai Institute of Applied Mathematics and Mechanics, School of Mechanics and Engineering Science, Shanghai University, Shanghai 200444, China

**Keywords:** two-phase flows, microfluidics, dripping-jetting transition, monodisperse droplet generation, coaxial flow

## Abstract

The formations of micro-droplets are strongly influenced by the local geometries where they are generated. In this paper, through experimental research, we focus on the roles of microchannel tapering in the liquid paraffin/ethanol coaxial flows in their flow patterns, flow regimes, and droplet parameters, i.e., their sizes and forming frequencies. For validity, the non-tapering coaxial flows (the convergence angle $\alpha =0^{\circ }$) are investigated, the experimental methods and experimental data are examined and analyzed by contrasting the details with previous works, and consistent results are obtained. We consider a slightly tapering microchannel (the convergence angle $\alpha =2.8^{\circ }$) and by comparison, the experiments show that the tapering has significant effects on the flow patterns, droplet generation frequencies, and droplet sizes. The regimes of squeezing, dripping, jetting, tubing, and threading are differentiated to shrink toward the coordinate origin of the $Ca_{c}$–$We_{d}$ space. The closer it is to the origin, the less variations will occur. For the adjacent regimes of the origin, i.e., dripping and squeezing, slight changes have occurred in both flow patterns, as well as the droplet characters. In the dripping and squeezing modes, the liquid droplets are generated near the orifice of the inner tube. Their forming positions (geometry) and flow conditions are almost the same. Therefore, the causes of minute changes in such regimes are physically understandable. While in the jetting regimes, the droplets shrink in size and their forming frequencies increase. The droplet sizes and the frequencies are both linearly related to those of the non-tapering cases with the corresponding relations derived. Furthermore, the threading and the tubing patterns almost did not emerged in the non-tapering data, as it seemed easier to form elongated jets, thinning or widening, in the tapered tubes. This can be explained by the stable analysis of the coaxial jets, which indicates that the reductions in the microchannel diameters can suppress the development of the interface disturbances.

## 1. Introduction

Microfluidic technology has many advantages, including the precise control of the droplet volume and manipulation of individual droplets, which saves raw materials, produces monodisperse droplets in large batches, and has a large specific surface area that facilitates rapid reactions [1]. Due to the advantages, microfluidic technology has been widely used for drug delivery [2], cell capsules, or digital PCR (polymerase chain reaction) systems [3,4,5,6], protein crystallization [7], polymer microcapsules [8], and microreactors [9,10].

The studies of flow patterns in gas–liquid or liquid–liquid two-phase flows in microchannels are the basis of two-phase fluid flow behaviours. The geometries of the microchannels are very important control elements for droplet generations. Commonly used microchannel geometries are T-shaped (Y-shaped) [11,12,13,14], cross-shaped [15,16], flow-focused [17], and coaxial [18,19,20,21,22]. The differences are in the flow orientations and the manners in which the continuous phase, with a flow rate $Q_{c}$, and the dispersed phase, with a flow rate $Q_{d}$, meet each other.

The literature [23] studied the relationship between the angles of the continuous and the dispersed phases in the cross-shaped microchannels, and found that the droplet generation frequencies were higher in a microchannel at a $90^{\circ }$ intersecting angle than that of a $30^{\circ }$ one. Researchers have long recognized the important role of geometric constraints in microfluidics [11,12,13,15,16,17]. The above are just a few typical geometries. Flows in each geometric structure have their own uniqueness and commonality.

In this paper, we mainly discuss the two-phase flow characteristics caused by the local tapering geometry of the coaxial microfluidics. The coaxial microchannels are nested structures, i.e., the inner tube opens into the dispersed phase and the outer annular tube opens into the continuous phase. The continuous phase and the dispersed phase flow in the same direction and meet at the orifice of the inner tube [19,21,24,25,26,27]. The dimensionless numbers, such as the capillary number $Ca_{c}=\eta_{c}u_{c}/\sigma$ of the continuous phase, the Weber number $We_{d}=\rho_{d}u_{d}^{2}D_{d}/\sigma$ of the dispersed phase, the Reynolds number $Re_{d}=\rho_{d}u_{d}D_{d}/\eta_{d}$ of the dispersed phase, and the ratio of the two-phase flow rates $Q_{d}/Q_{c}$, are generally used to describe these physical systems. Where η, u=Q/A, σ, ρ, *A* and *D* are, respectively, the dynamic viscosity, the average velocity, the density, the interfacial tension, the flow cross-sectional area, and the diameter, with subscripts “*c*” and “*d*” representing the continuous phase and the dispersed phase.

The capillary number is a measure of the relative importance of viscous force compared with interfacial tension. The Weber number is used to measure the relative importance of the inertial force to the interfacial tension. The capillary number and the Weber number are well-known parameters to distinguish the flow patterns in microfluidics. The Reynolds number is used to measure the relative importance of inertial force and viscous force, which is small ($Re_{d}<O\left(1\right)$) to indicate that the microchannel flow is laminar.

In the microfluidic research, monodisperse droplet generation is the focus of attention. The flow pattern of the liquid, the size of the generated droplets, and the frequency of generation are also very important. Squeezing, Dripping, and Jetting modes are the patterns that can produce monodisperse droplets, which are mostly investigated as two-phase flow patterns in microchannels [26,28,29,30]. The inherent physical mechanisms of these modes for generating monodisperse droplets are different. The Squeezing mode occurs when the flow rate of the two-phase fluid is relatively small, and when the flow rate of the dispersed phase is relatively large, the capillary number is also relatively small ($Ca_{c}<0.01$).

The dispersed phase accumulates at the nozzle and the resulting liquid mass impedes the continuous phase flow, causing an increase in the upward-downward pressure difference of the dispersed bulk. The incoming stream continuously squeezes the liquid mass to flow downward until the pressure difference overwhelms the surface tension force, and the dispersed phase micelles depart from the upstream dispersed jet to form large droplets [31,32,33]. When the flow rate of the continuous phase is relatively fast, the dispersed phase fluid is subjected to the viscous shearing force of the continuous phase, and after the interfacial tension is overcome, monodisperse droplets are formed at the nozzle outlet, which is the Dripping mode ($0.01<Ca_{c}<0.1$). The size of the droplets formed is close to and slightly below the tube diameter. As the capillary number continues to increase ($Ca_{c}>0.1$), a jet forms, and the droplets are not formed near the exit of the inner tube, but at the rather downstream jet end, owing to the shear stretching of the outer flow. When the jet length is more than three times the inner tube diameter, it is categorized as the Jetting mode. This pattern occurs only when the flow rate of the continuous phase and its viscosity are relatively large [26,28,34].

In the study of these patterns, researchers have discovered some physical laws. For example, the droplet diameters in the Dripping mode are inversely proportional to the continuous phase capillary number $Ca_{c}$, while the droplet diameters in the Jetting mode are proportional to the 1/2 power of the flow rate ratio, $Q_{d}/Q_{c}$ [29,35]. In more cases, the laws that are found are not necessarily consistent and thus require careful and meticulous researches. Boundary constraints are always one of the most important directions in digital microfluidics.

In our previous work, [36], we proposed improvement of the control characteristics of bubble generation by tapering the microchannels, and tested our judgements through numerical simulations. We discovered a new mechanism of mixed influences about ‘stretching’ under coaxial shearing with ‘squeezing’ through upstream phase holdups in T-shaped junctions. We found that the tapering configuration can increase the bubbling frequency and decrease the sizes of bubble/droplet, exponentially.

In this paper, we want to observe the actual effects of tapered microchannels on droplet forming through experiments. Two coaxial microchannels, with convergence angles $\alpha =0^{\circ }$ and $\alpha =2.8^{\circ }$ (limited to manufacturing conditions), respectively, are adopted. Through experiments, the variation tendencies of the flow patterns and the droplet forming characteristics during to the convergence angle changes are studied.

## 2. Materials and Experimental Set-Ups

Liquid paraffin and ethanol are adopted as the working fluids. The detailed material properties, dynamic viscosity η, density ρ, and liquid paraffin–ethanol interfacial tension σ, are depicted in (Table 1). Both densities are quite close, while the liquid paraffin is ten-times more viscous compared to ethanol.

**Figure 1:**
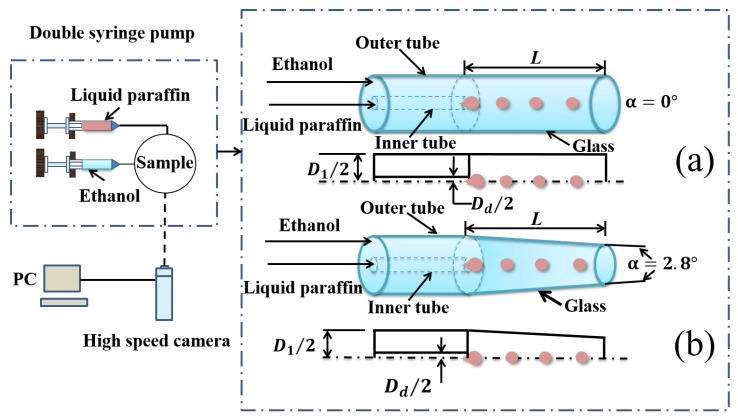
A schematic diagram of the experimental set-ups. The inner liquid paraffin is injected into the coaxial annular ethanol jet in a nested round microchannel. This results in the breakups of liquid paraffin into droplets in the downstream. Two convergence angles of the outer tapered tube are considered. (**a**) $\alpha =0^{\circ }$, (**b**) $\alpha =2.8^{\circ }$.

The two immiscible liquids, driven by a high-precision, two-channel injection pump (JZB1800D, JY, China), are injected into a nested coaxial outer-inner annular microchannel, as shown in Figure 1, with the inner steel needle extending into the outer quartz tube. The inner diameters of the outer tube and the inner tube, respectively, are 1.26 mm and 0.26 mm. The outer tube is 18.30 mm longer than the inner tube. The dispersed phase (liquid paraffin), through the inner steel needle, is injected into the surrounding annular continuous phase (ethanol) circled by the the outer tube. The difference between Figure 1a,b is the convergence angles of the outer quartz tube.

In Figure 1a, the structure is quite normal parallel ($\alpha =0^{\circ }$) annular jets. In Figure 1a, the outer quartz tube is tapered with a slight convergence angle ($\alpha =2.8^{\circ }$). The outer tube is quite wettable with the continuous ethanol phase. The tapering glass micro-tubes were made by stretching straight ones while heating. It is hard to derive a perfect conical inner tube wall and achieve a well-defined and well-measured tapering angle in this process. Meanwhile, the axial stretching did not allow a taping angle large enough. We had picked one of the least problematic tubes, as shown in this research. Quartz glass was adopted to make the tube convenient to observe.

A high-speed camera (Phantom V611-16G-M, AMETEK, USA) is used, with a micro-lens (AT-X M100 PRO-D, Tokina, Japan), to capture images of the two-phase flow. All data are real-time stored in the computer. The two-phase interfacial tension coefficient σ is measured using the pendant drop method [37]. The instrument is an interfacial tension meter (SL200KS, Kinσ, America). The fluid dynamic viscosity η is measured by a rotational rheometer (RS6000, HAAK, Germany).

**Figure 2:**
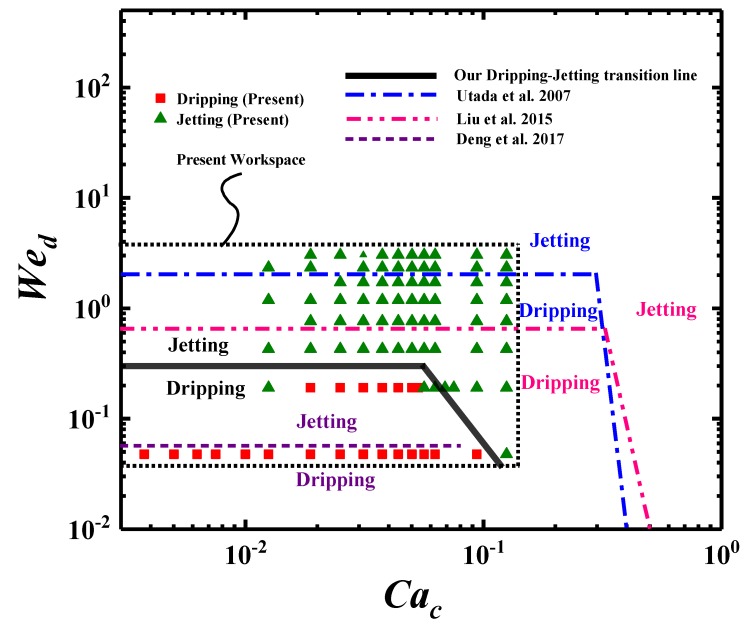
Comparisons of Dripping and Jetting on the flow pattern regions in ($Ca_{c}$, $We_{d}$) space.

During the present experiments, the dispersed phase (liquid paraffin) flow rate range is $Q_{d}$ = 6–48 mL/h, and the continuous phase (ethanol) flow rate range is $Q_{c}$ = 12–600 mL/h. Within these flow rates, the Weber number, $We_{d}$, varies between 0.01 and 0.2, and the capillary number, $Ca_{c}$, varies between 0.06 and 0.1, as shown in Figure 2. It should be noted that the existing orifice about the present experiment setting was other than most microchip channel ones, which was designed for generating monodisperse particles or dispensing compound materials. In fact, they were in the practical usage of inkjet-printing and additive manufacturing. There should exist the influence of external flows, which is out of scope of the present work.

## 3. Liquid Paraffin–Ethanol Coaxial Flow at the Convergence Angle $\alpha =0^{\circ }$

### 3.1. State Diagram about the Dripping-to-Jetting Transitions

Many different flow patterns of forming droplets, known as Squeezing, Dripping, and Jetting, are observed in the microchannel. We compare the state diagram of our results with those of the experiments [22,27,38]. The transition between Dripping and Jetting has always been the key subject. In Figure 2, we give the comparisons of state diagrams as a function of $Ca_{c}$ and $We_{d}$. We note the slightly different microchannels used in the present work. The streams in [22,27,38] are all two-phase coaxial inner flow in microchannels with theoretically unlimited extensions. While there is an orifice at the the end of our outer tube, we only concentrate on the inner flows here.

In Figure 2, the data of the present work are in the dashed frame. The filled ’square’ and filled ’triangle’, respectively, represent our Dripping and Jetting modes. The lines represent the Dripping-to-Jetting transition boundaries. We find that the physical figures of dripping-to-jetting transitions are highly consistent in the Cac Wed space among our results and others’ work [22,27,38]. However, the exact positions of the transition lines have differences of nearly two orders of magnitude from all of these studies, at the same bases of characteristic parameters.

The Weber number $We_{d}$, of the Dripping-to-Jetting transition horizontal lines, is in the range of approximately 0.03 to 2. Our result is $We_{d}=0.3$ in the qualitative region between the results of Deng et al. [27] with Liu et al. [22] and Utada et al. [38]. The corresponding capillary number $Ca_{c}$ is in the range of $10^{-3}$–$10^{-1}$. The value differences among these works are usually due to inconsistencies in the experiment setups and data treatments. The horizontal lines indicate that when the continuous phase capillary number $Ca_{c}$ is small, the Dripping-to-Jetting transition is mainly controlled by the dispersed phase Weber number $We_{d}$, and the interfacial tension force is the main driving force. The jetting may happen by decreasing the interfacial tension force. For the declined lines at larger $Ca_{c}$, the values also have differences though the tendencies are alike.

### 3.2. Droplet Diameter in Dripping Mode

**Figure 3:**
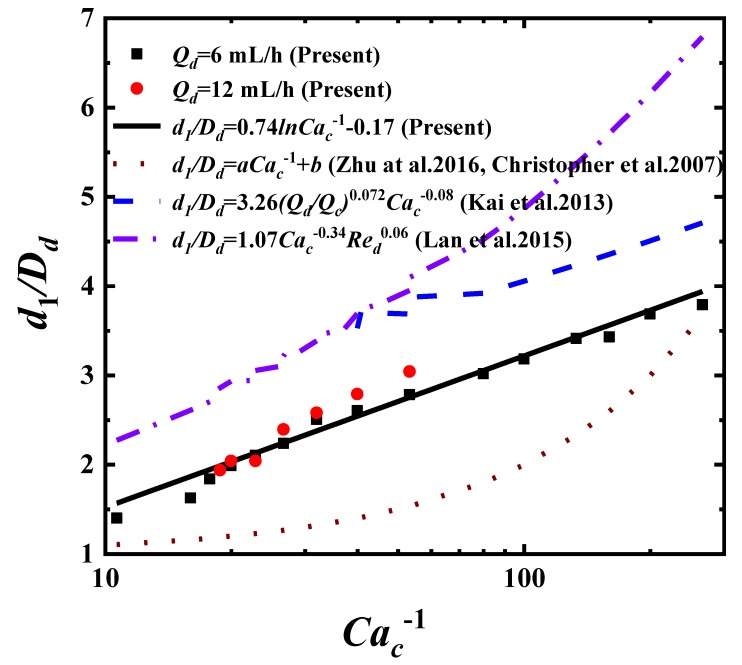
The normalized droplet diameter $\frac{d_{1}}{D_{d}}$ as function of the continuous phase capillary number $Ca_{c}^{-1}$ in the Dripping mode.

The Dripping mode is the main flow pattern that generates uniform-sized, strict monodispersed droplets. Studies have shown, during the different droplet formation mechanisms or detailed aspects, that empirical formulas of Dripping for droplet characteristics vary from those of Jetting and Squeezing [25,26,39,40]. Dripping happens under moderate flow rates and pinches off not far away from the inner needle orifice. The interfacial tension dominates the droplet growing process. The pinch-off occurs when the viscous shear force from the continuous phase overcomes the interfacial tension force at a critical droplet volume, and the interfacial tension force can not hold the droplet attached to the needle tip. The ratio between the viscous shear force and the interfacial tension force is described by capillary number $Ca_{c}$, the corresponding pinch-off critical capillary number is $Ca_{c}∼O\left(1\right)$. The droplet diameters in the Dripping mode should be a function of $Ca_{c}$, as testified by many works [25,26,39,40]. Our data of droplet diameters fits

(1) $$\frac{d_{1}}{D_{d}}=0.74lnCa_{c}^{-1}-0.17,$$

where the droplet diameter $d_{1}$ is normalized by the needle inner diameter $D_{d}$. This result is compared at flow rates $Q_{d}$ = 6 mL/h and $Q_{d}$ = 12 mL/h, as shown in Figure 3, with the references [25,26,39,40]. All these formulas are the function of $Ca_{c}^{-1}$, as wished, except we take the $lnCa_{c}^{-1}$ form. Though the referred results seem to have little consistency on the fitting curves and are different in the formulas, our results are shown to be in the middle ranges of those data. This may be caused by many detailed aspects, such as the contact and wetting properties between phases, the thickness of the needle wall, or the underdeveloped velocity profiles adjacent to the inner needle tip.

### 3.3. Droplet Diameter in the Jetting Mode

The Jetting mode is characterized by a long jet emitting droplets downstream, and the continuous phase viscous shear force dominates the Jetting process. The droplet finally pinches off and departs from the jet tip owing to Rayleigh–Plateau instability [41,42]. Irregular satellite droplets may form by nonlinear wave fluctuations at the jet interface.

In the Jetting mode, the droplet diameter is greatly affected by the two-phase flow rates, strictly, the velocity difference. Following the clue given in [35], for a low-Reynolds number microchannel flow, the stream is in a steady-state. The Stokes equation,

(2) $$\nabla p_{i}=\eta_{i}▵u_{i}$$

is used to describe such a flow for both phases. Subscript $i{=}^{\prime }c^{\prime }{,}^{\prime }d^{\prime }$ represents, respectively, the continuous phase and the dispersed phase. We denote $D_{1}$ as the inner diameter of the outer tube, $d_{jet}$ as the diameter of the long jet, $d_{1}$ as the droplet diameter, *p* as the pressure, and define $s=\frac{d_{jet}}{D_{1}}$. The solution of Equation (2) derives as [35]

(3) $$\frac{Q_{d}}{Q_{c}}=\frac{\eta_{c}}{\eta_{d}}\frac{s^{4}}{{\left(1-s^{2}\right)}^{2}}+\frac{2s^{2}}{1-s^{2}}.$$

According to the Rayleigh–Plateau instability of jetting, when the wavelengh of the fastest growing disturbance exceeds the jet circumference, a wavelengh volume of the jet will pinch off and form a droplet at the jet tip by increasing the spatial wave. Therefore, the size of the droplet $d_{1}=ad_{jet}$ (a is a constant). The solution of Equation (3) is

(4) $$s^{2}=\frac{-\sqrt{\frac{\eta_{c}}{\eta_{d}}\frac{Q_{d}}{Q_{c}}+1}+\frac{Q_{d}}{Q_{c}}+1}{-\frac{\eta_{c}}{\eta_{d}}+\frac{Q_{d}}{Q_{c}}+2}.$$

For $\frac{\eta_{c}}{\eta_{d}}=1$ and $\frac{Q_{d}}{Q_{c}}\ll 1$, Equation (4) transfers into

(5) $$\begin{array}{cc} s^{2} & =1-\frac{1}{\sqrt{\frac{Q_{d}}{Q_{c}}+1}}=1-\left(1-\frac{1}{2}\frac{Q_{d}}{Q_{c}}+O{\left(\frac{Q_{d}}{Q_{c}}\right)}^{2}\right)\\ \; & ≐\frac{1}{2}\frac{Q_{d}}{Q_{c}}. \end{array}$$

**Figure 4:**
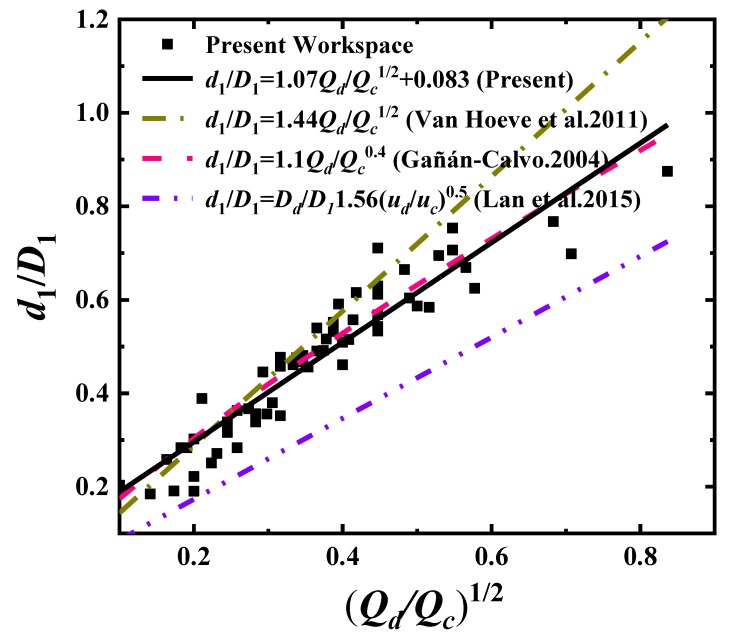
Fitting data of the normalized droplet diameter $\frac{d_{1}}{D_{1}}$ as a function of ${\left(\frac{Q_{d}}{Q_{c}}\right)}^{0.5}$.

Hence,$\frac{d_{1}}{D_{1}}=a\frac{d_{jet}}{D_{1}}∼s∼{\left(\frac{Q_{d}}{Q_{c}}\right)}^{1/2}$, as most studies derive when fitting the droplet diameters. In Figure 4, we plot and compare the fitting data for the normalized droplet diameter $\frac{d_{1}}{D_{1}}$ as a function of ${\left(\frac{Q_{d}}{Q_{c}}\right)}^{1/2}$. Our formula is

(6) $$\frac{d_{1}}{D_{1}}=1.07{\left(\frac{Q_{d}}{Q_{c}}\right)}^{0.5}+0.083\approx 1.07{\left(\frac{Q_{d}}{Q_{c}}\right)}^{0.5}.$$

It can be seen that, regardless of $\frac{\eta_{c}}{\eta_{d}}\approx 0.08\ll 1$ in the experiments, our results show good consistency also with the other studies [19,26,38], and the exponential power 1/2 of the flow rate ratio still holds as well.

## 4. The Role of Convergence Angle, $\alpha =0^{\circ }$ and $\alpha =2.8^{\circ }$

### 4.1. The Influence of Microchannel Tapering on the State Diagram

In digital microfluidics, the geometry of the microchannel takes significant roles in two-phase flow states and droplet generation characteristics. Here, we discuss the effects caused by tapering the outer tube of the microchannel. Two comparative convergence angles, $\alpha =0^{\circ }$ (non-tapered) and $\alpha =2.8^{\circ }$, are considered. The relative variations in the characteristics of the liquid paraffin/ethanol microchannel flow will be observed. The ranges of the experimental parameters are as forementioned: The dispersed phase (liquid paraffin) flow rate $Q_{d}$ = 6–48 mL/h, and the continuous phase (ethanol) flow rate $Q_{c}$ = 12–600 mL/h. Several hundreds of experiments are performed in the two convergence angles, $\alpha =0^{\circ }$ and $\alpha =2.8^{\circ }$, tubes.

**Figure 5:**
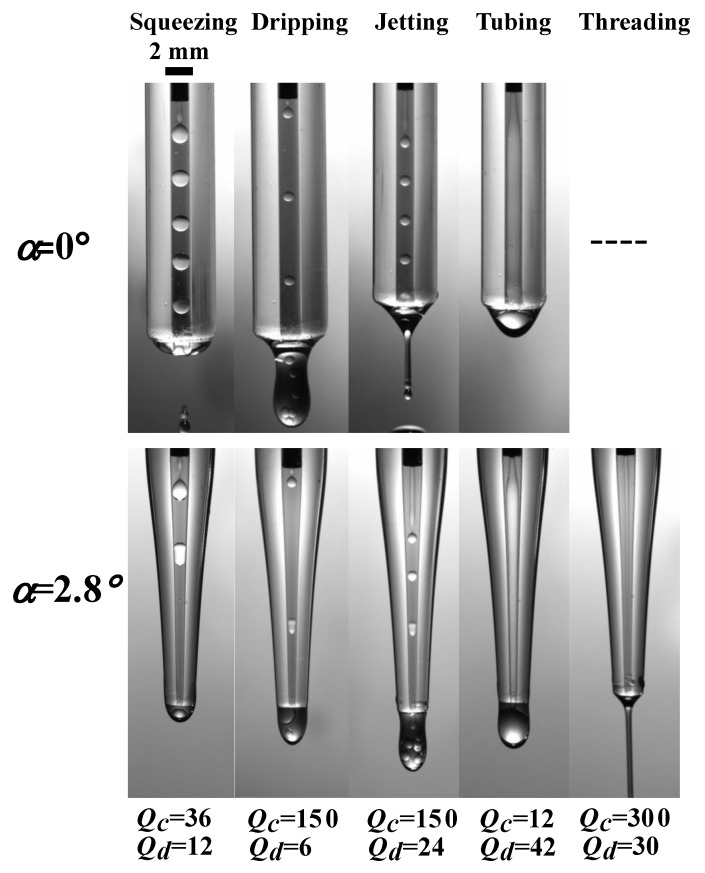
Five flow patterns occurring in coaxial microchannels with convergence angles $\alpha =0^{\circ }$ and $\alpha =2.8^{\circ }.$

We select the typical flow patterns at the two convergence angles on the same parameters, ((a) $Q_{c}=36,Q_{d}=12$; (b) $Q_{c}=150,Q_{d}=6$; (c) $Q_{c}=150,Q_{d}=24$; (d) $Q_{c}=12,Q_{d}=42$; (e) $Q_{c}=300$, $Q_{d}=30$), and plot in a up-down couple style, as shown in Figure 5. The corresponding flow patterns, respectively, are Squeezing, Dripping, Jetting, Tubing, and Threading. These flow states are well discussed in the literature [25,26,35,42]. We will mainly focus on the variations coming from microchannel tapering.

**Figure 6:**
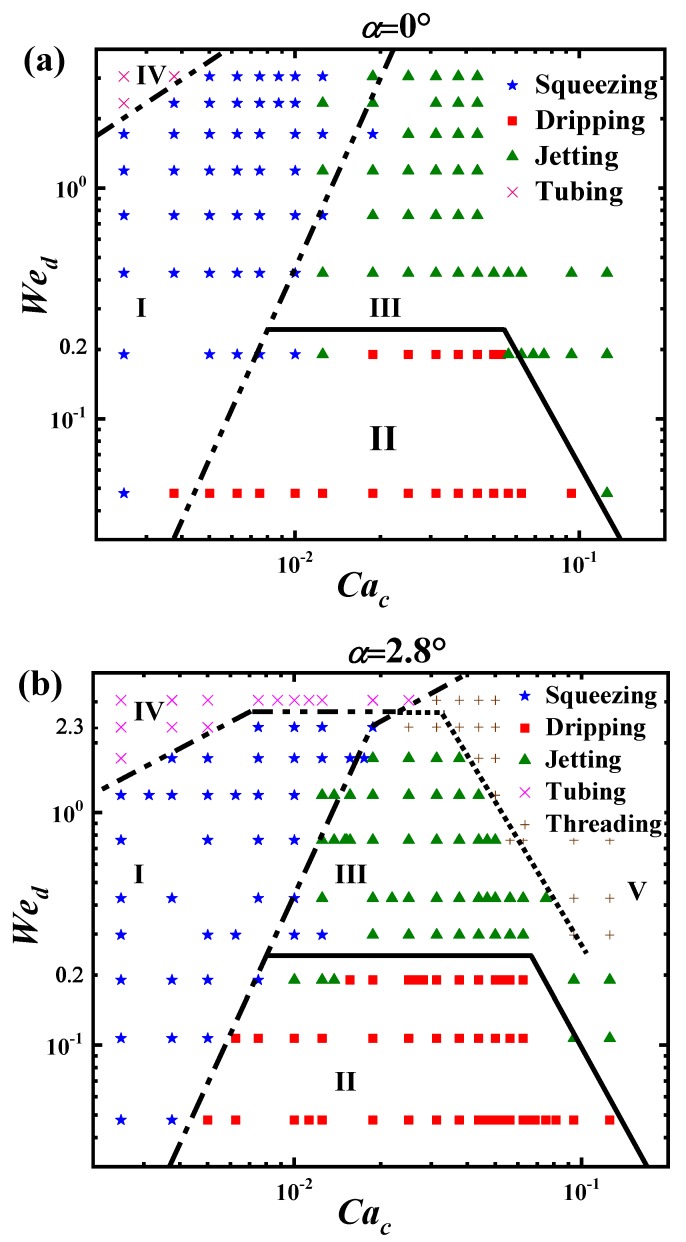
State diagrams in the $Ca_{c}$–$We_{d}$ space at the two convergence angles. (**a**) $\alpha =0^{\circ }$ and (**b**) $\alpha =2.8^{\circ }.$

The state diagrams for each convergence angle are plotted in Figure 6. The separating lines are added to discriminate between the modes, Squeezing (I), Dripping (II), Jetting (III), Tubing (IV) and Threading (V). The Squeezing (I) region is in the low $Ca_{c}$ area, the inertia of the dispersed phase and the surface tension force dominate this process, and produce droplets bigger than the tube diameters. The Dripping (II) region is in the low $We_{d}$ area and moderate $Ca_{c}$ area, and the viscous shear force of the continuous phase and the interface tension force dominate this process, and produce droplets of sizes comparative to the inner diameter of the outer tube $D_{1}$.

The Jetting (III) region is in the moderate $We_{d}$ area and moderate $Ca_{c}$ area, and the viscous shear force dominates this process, and produces droplets much smaller than the inner diameter of the outer tube $D_{1}$. The Tubing (IV) region is in the big $We_{d}$ area and low/moderate $Ca_{c}$ area, as the velocity of the dispersed phase is relative high. The Threading (V) region is in the big $Ca_{c}$ area, as the velocity of the continuous phase is relatively much higher.

From Figure 6, comparing to state diagram of $\alpha =0^{\circ }$, there are obvious variations about the transition lines between the districts at the two convergence angles ($\alpha =0^{\circ }$ (upper) and $\alpha =2.8^{\circ }$ (lower)). Accurately, the changes happen mainly in big $Ca_{c}$ and/or big $We_{d}$ areas. The Squeezing-to-Dripping transition line (dash-dot), the Squeezing-to-Jetting transition line (dash-dot), and the Dripping-to-Jetting transition line(solid) change little, while the Squeezing-to-Tubing transition line (dash-dot-dot) goes down to compress the Squeezing region in the low $Ca_{c}$ area and the Jetting region in the moderate $Ca_{c}$ area.

The largest change is the emergence of the Jetting-to-Threading transition line (dot), which suppresses the Jetting region greatly at large $Ca_{c}$ and large $We_{d}$. Such an emergence is also observed in Figure 5 at ($Q_{c}=300,Q_{d}=30$). We cannot find the comparable plot in the data of the straight tube ($\alpha =0^{\circ }$). Hence, for the modes of forming droplets, i.e., Squeezing, Dripping, and Jetting, the change of the convergence angle from ($\alpha =0^{\circ }$) to ($\alpha =2.8^{\circ }$) mostly affects the Jetting area and has no significant role on the Squeezing and Dripping modes.

### 4.2. The Influence of Microchannel Tapering on Droplet Generation Characteristics in the Dripping and Jetting Modes

#### 4.2.1. Dripping Mode

**Figure 7:**
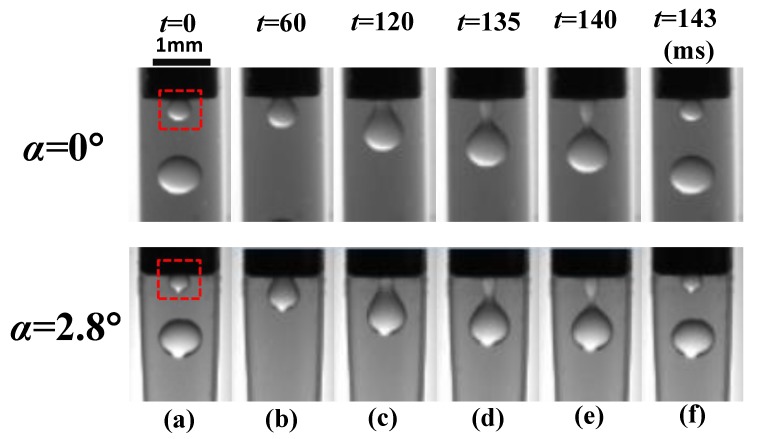
The formation process (one period) of a paraffin droplet under different convergence angles in the Dripping mode.

When the microchannel convergence angle is of $\alpha =0^{\circ }$ and $\alpha =2.8^{\circ }$, the formation of paraffin droplets in the Dripping mode is illustrated in Figure 7 at ($Q_{d}=6$, $Q_{c}=150$), in time sequences (*t* = 0, 60, 120, 135, 140, and 143 ms). The decrease of time increments is owing to the unevenness of the droplet forming process. Seen from Figure 7, the formation of paraffin droplets is mainly divided into three stages: the growing stage (a–c), the necking stage (c–e), and the pinch-off stage (e–f). The droplet growing takes the most time (above 80%) in a period, the necking processes around 10%, and the pinch-off is lower than 5%.

**Figure 8:**
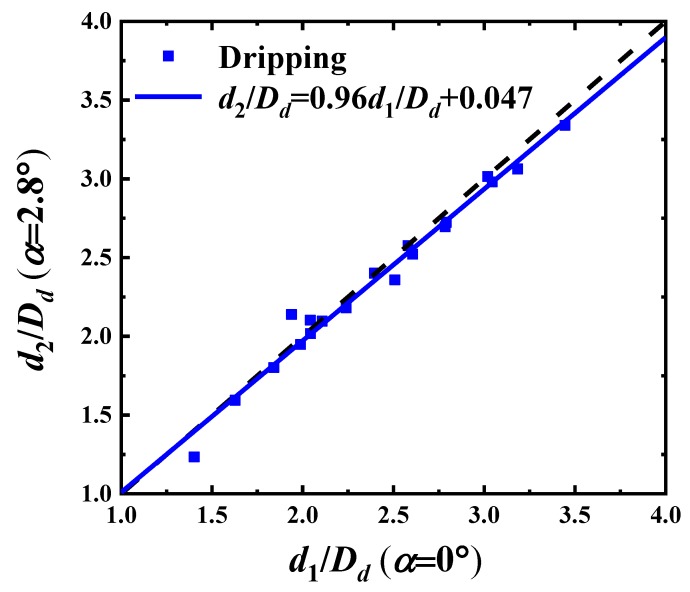
The correlation test of paraffin droplet sizes between $\frac{d_{1}}{D_{d}}$ for $\alpha =0^{\circ }$ and $\frac{d_{2}}{D_{d}}$ for $\alpha =2.8^{\circ }$.

**Figure 9:**
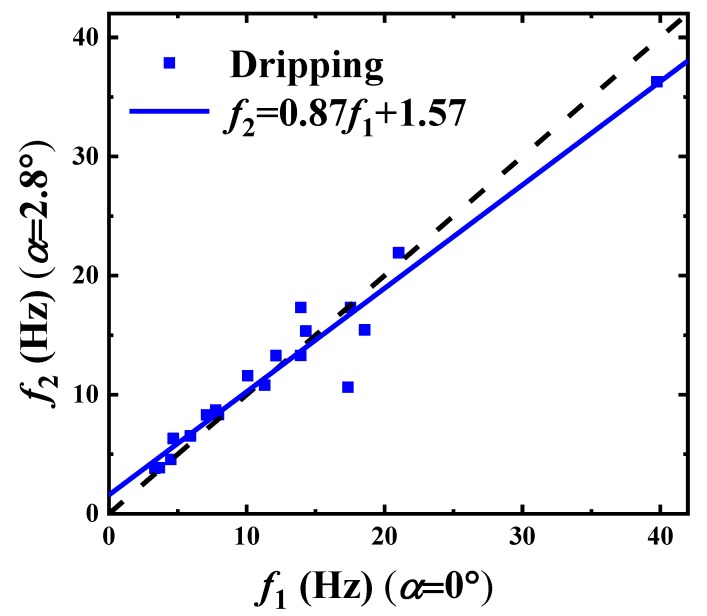
The correlation test of paraffin droplet forming frequencies between $f_{1}$ for $\alpha =0^{\circ }$ and $f_{2}$ for $\alpha =2.8^{\circ }$.

By comparing the upper $\alpha =0^{\circ }$ and the downward $\alpha =2.8^{\circ }$ slides in Figure 7, the morphologies of droplet forming of the two angles hardly vary between each other as time passes. It can be foreseen from the upstream geometrical and flow conditions, where both the paraffin droplets are generated near the tip of the inner needle. The tapering region is not started to take effects. Therefore, it is reasonable that the convergence angle has no obvious effect on the formation of the paraffin droplets in the Dripping mode, which is consistent with the state diagram we derived in the above section. It can also be testified by checking on the droplet sizes (Figure 8) and forming frequencies (Figure 9).

It can be seen from Figure 8 that we have $\frac{d_{2}}{D_{d}}=0.96\frac{d_{1}}{D_{d}}+0.047$. The droplet size deviation between the two experiments is too small to be counted, as well as that of the frequency of droplet generation in Figure 9 for the dripping frequency $f_{2}=0.87f_{1}+1.57$. As Figure 8 and Figure 9 show, both the slope of the droplet diameter ratio $\frac{d_{2}}{d_{1}}$ and the frequency ratio $\frac{f_{2}}{f_{1}}$ are close to 1, which indicates that the convergence angle does not react severely under such cases. Otherwise, the existing variation also indicates that the frequency is easier to be influenced by the tapering than the droplet size through comparing the fitting coefficients, 0.96 and 0.87.

#### 4.2.2. Jetting Mode

**Figure 10:**
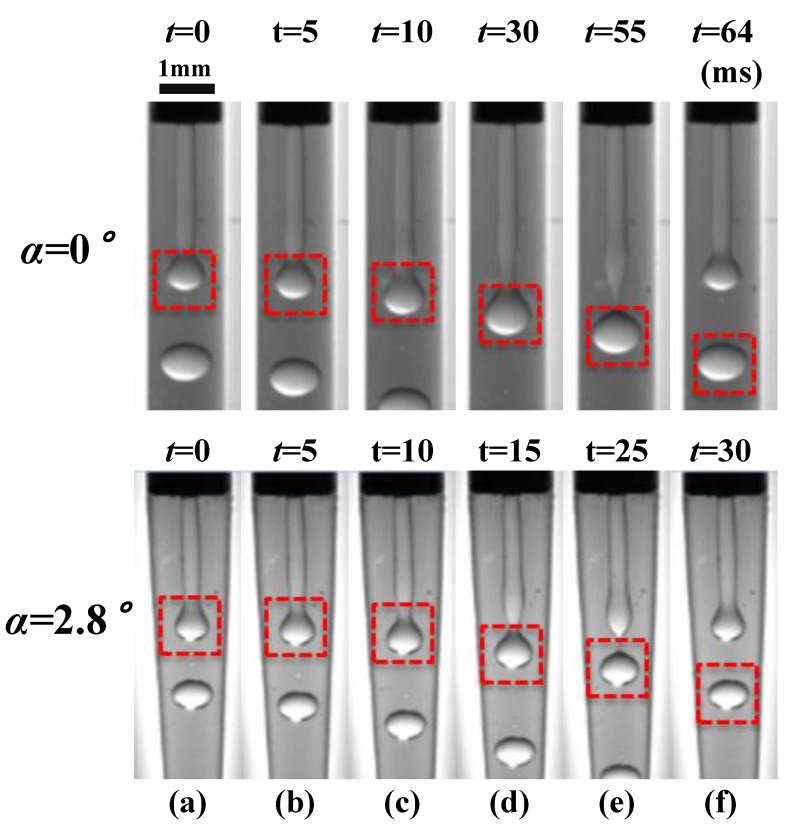
The formation process (one period) of a paraffin droplet under different convergence angles in the Jetting Mode.

A typical paraffin droplet formation in the Jetting mode is shown in Figure 10 for $Q_{c}$ = 150 mL/h and $Q_{d}$ = 30 mL/h, independently, in a microchannel of convergence angle $\alpha =0^{\circ }$ (upper) or $\alpha =2.8^{\circ }$ (lower). The time sequences of the period are t=0,5,10,30,55, and 64 ms for $\alpha =0^{\circ }$ and *t* = 0, 5, 10, 15, 25, and 30 ms for $\alpha =2.8^{\circ }$, respectively. The period can also be divided into three stages, the growing stage (a–c), the necking stage (c–e), and the pinch-off stage (e–f), as shown in Figure 10, similar to that in the dripping mode. The difference is that the paraffin droplets are formed far downstream away from the inner needle tip and the Rayleigh–Plateau instability dominates.

**Figure 11:**
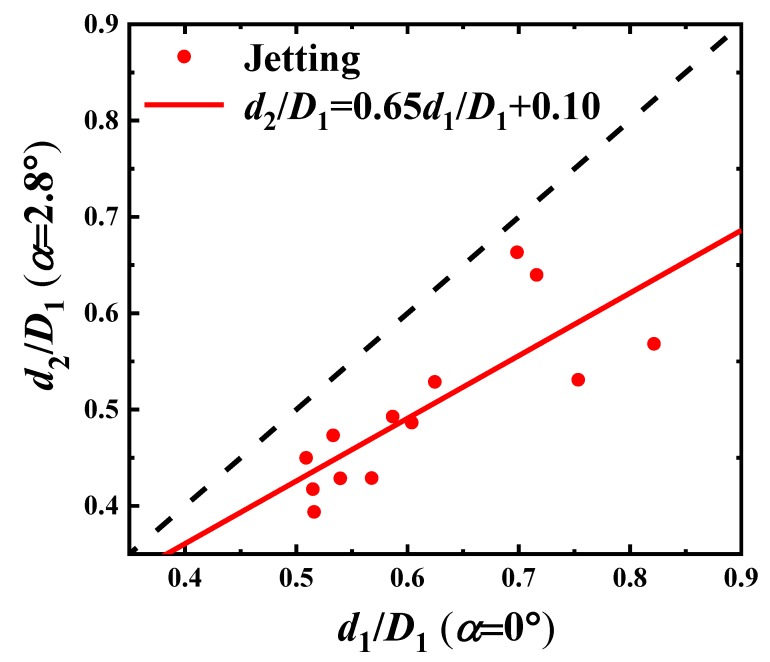
The correlation test of the paraffin droplet sizes between $\frac{d_{1}}{D_{d}}$ for $\alpha =0^{\circ }$ and $\frac{d_{2}}{D_{d}}$ for $\alpha =2.8^{\circ }$ in the Jetting Mode.

**Figure 12:**
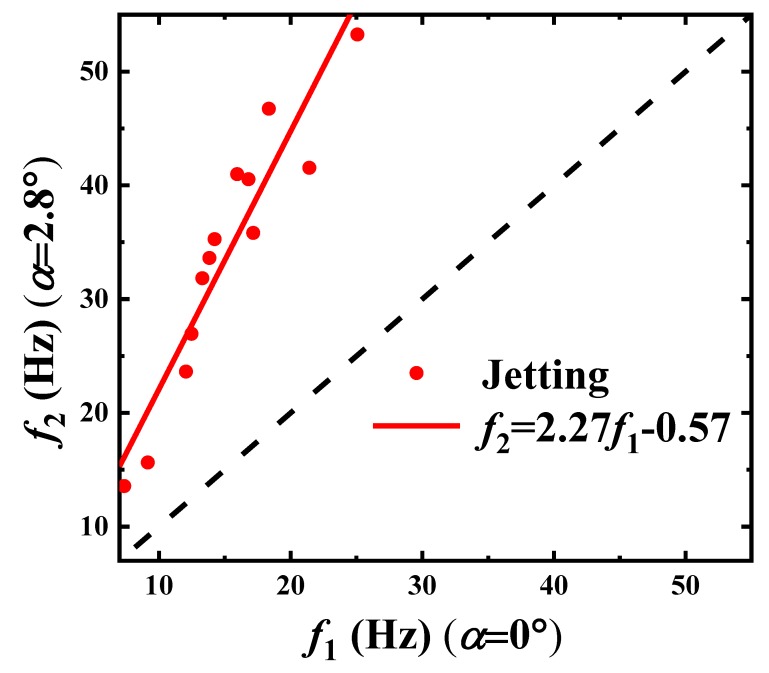
The correlation test of the paraffin droplet forming frequencies between $f_{1}$ for $\alpha =0^{\circ }$ and $f_{2}$ for $\alpha =2.8^{\circ }$ in the Jetting Mode.

In Figure 10, the period of droplet forming is shorter and the droplet is smaller in a tapering microchannel. The correlations of droplet size and droplet forming frequency for the two convergence angles $\alpha =0^{\circ }$ and $\alpha =2.8^{\circ }$ are plotted, separately, in Figure 11 and Figure 12.

We find linear relations between the two convergence angles, respectively. For the sizes of droplet, as shown in Figure 11, this satisfies

(7) $$\frac{d_{2}}{D_{1}}=0.65\frac{d_{1}}{D_{1}}+0.10.$$

For the droplet forming frequencies, as shown in Figure 12, we derive

(8) $$f_{2}=2.27f_{1}-0.57.$$

The slope of the droplet diameter ratio $\frac{d_{2}}{d_{1}}$ is 0.65, as shown in Equation (7), and that of the frequency ratio $\frac{f_{2}}{f_{1}}$ is 2.27, as shown in Equation (8). This shows the decrease in the droplet size and the increase in the droplet forming frequency, when increasing the convergence angle.

In fact, $\alpha =2.8^{\circ }$ is a very small tapering angle, and the stream in such a microchannel is nearly parallel. Assume that Equation (4) holds at every cross-section and axial location *x*, of the tapered tube. The diameter of cross-section at *x* is $\bar{D}\left(x\right)$, the diameter of jet $\bar{d_{jet}}\left(x\right)$ is approximated as

(9) $$\bar{d_{jet}}\left(x\right)=\sqrt{\frac{-\sqrt{\frac{\eta_{c}}{\eta_{d}}\frac{Q_{d}}{Q_{c}}+1}+\frac{Q_{d}}{Q_{c}}+1}{-\frac{\eta_{c}}{\eta_{d}}+\frac{Q_{d}}{Q_{c}}+2}}\bar{D}\left(x\right)∼\bar{D},$$

and the derivative of the jet diameter to cross-section change goes to

(10) $$\frac{\partial \bar{d_{jet}}}{\partial \bar{D}}=\sqrt{\frac{-\sqrt{\frac{\eta_{c}}{\eta_{d}}\frac{Q_{d}}{Q_{c}}+1}+\frac{Q_{d}}{Q_{c}}+1}{-\frac{\eta_{c}}{\eta_{d}}+\frac{Q_{d}}{Q_{c}}+2}}.$$

Clearly, $\frac{\partial \bar{d_{jet}}}{\partial \bar{D}}$ is greater than zero in our experiments, and varies as a function of the viscosity ratio and flow rate ratio, which can explain that the tapering thins the jet and decreases the size of the droplet, for droplet diameters $d_{2}∼\bar{d_{jet}}$. Following this line of thought, according to the Rayleigh–Plateau instability, the most unstable wave dominates the size of the droplet. The droplet forming frequency, $\bar{f}$ at *x*, can be expressed as

(11) $$\bar{f}=\frac{\bar{u}}{\pi \bar{d_{jet}}}=\frac{4Q_{d}}{\pi^{2}{\bar{d_{jet}}}^{3}}∼{\bar{D}}^{-3}.$$

where $\bar{u}=\frac{4Q_{d}}{\pi {\bar{d_{jet}}}^{2}}$ is the mean velocity of the jet for the dispersed phase. This can explain that the jetting frequency grows fast with the shrinking of the tube diameter.

## 5. Conclusions

The behaviours of paraffin/ethanol coaxial flow are considered by experiments, and the property variations of droplet formation corresponding to the change of convergence angle from $\alpha =0^{\circ }$ to $\alpha =2.8^{\circ }$ are investigated. The following conclusions are obtained:

(1) In the straight-tube ($\alpha =0^{\circ }$) coaxial stream, the experimental results of the Dripping-to-Jetting transition, flow patterns, and droplet characteristics are consistent with the data and fitting laws in the related literature.
(2) The tapered microchannel ($\alpha =2.8^{\circ }$) changes the state diagram of the liquid paraffin/ethanol coaxial in the $Ca_{c}$–$We_{d}$ space. The Dripping mode is not affected, and the Jetting regime is the most compressed by the emerging Threading mode at a large $Ca_{c}$.
(3) The tapered microchannel ($\alpha =2.8^{\circ }$) causes little change about the droplet forming processes in the Dripping mode, which is different from that of the Jetting mode. In the Jetting mode, the size of the droplet decreases as the tube diameter reduces by tapering, which is exactly the opposite for the droplet forming frequency. The droplet forming frequency grows quickly as the tube diameter reduces, $∼{\bar{D}}^{-3}$.

Interestingly, both the droplet size and the droplet forming frequency, obtained at convergence angle $\alpha =2.8^{\circ }$, are linearly proportional to those of the droplet derived in the straight tube ($\alpha =0^{\circ }$).

The mechanisms of such behaviours have not been well studied so far. Although, in some cross-focusing and flow-focusing microfluidic studies, researchers have considered the shear-induced thinning effect of the jet flow. The questions about how the boundary constraints affects the jet interface and causes flow instability have not been carefully discussed. There are still relatively open issues to be explored.

## Figures and Tables

**Table 1:** The physical properties of the two-phase flow (room temperature $22{\;}^{\circ }$ C).

Phase	Material	η (mPa·s)	ρ (g/cm ${}^{3}$)	σ (mN/m)
Continuous phase	Ethanol (95 vol.%)	1.3	0.817	4.5
Dispersed phase	Liquid paraffin	15.8	0.836
